# Supplementary material for: Effects of Extraction Technique on the Content and Antioxidant Activity of Flavonoids from Gossypium Hirsutum linn. Flowers
Source: Molecules. 2022 Aug 31;27(17):5627. doi: 10.3390/molecules27175627 (PMC9458133; doi:10.3390/molecules27175627)
Supplement: Supplementary file 1 [file molecules-27-05627-s001.zip › molecules-1871147-supplementary.pdf]

Supplementary material

Supplementary Table S1. Experimental Design Data of RSM Optimization

	Extraction time	Extraction temperature	Liquid ratio	Ethanol concentration
-1	1.0 h	45℃	1:15	65%
0	1.5 h	50℃	1:20	70%
1	2.0 h	55℃	1:25	75%
